# Percolating hierarchical defect structures drive phase transformation in Ce_1−*x*_Gd_*x*_O_2−*x*/2_: a total scattering study

**DOI:** 10.1107/S2052252515011641

**Published:** 2015-07-30

**Authors:** Marco Scavini, Mauro Coduri, Mattia Allieta, Paolo Masala, Serena Cappelli, Cesare Oliva, Michela Brunelli, Francesco Orsini, Claudio Ferrero

**Affiliations:** a Dipartimento di Chimica, Università di Milano, via C. Golgi 19, Milano I-20133, Italy; b Istituto di Scienze e Tecnologie Molecolari, CNR-ISTM, Milano I-20133, Italy; c Istituto per l’Energia e le Interfasi, CNR-IENI, C.so Promessi Sposi 29, Lecco I-23900, Italy; d SNBL/ESRF, 71 Avenue des Martyrs, CS 40220, Grenoble Cedex 9, 38043, France; e Dipartimento di Fisica, Università di Milano, Via G. Celoria 19, Milano I-20133, Italy; f ESRF – The European Synchrotron, 71 Avenue des Martyrs, CS 40220, Grenoble Cedex 9, 38043, France

**Keywords:** doped ceria, disorder, pair distribution function, high-resolution X-ray powder diffraction, percolation, hierarchy, solid electrolytes, electron spin resonance

## Abstract

A new hierarchical approach is presented for elucidating the structural disorder in Ce_1−*x*_Gd_*x*_O_2−*x*/2_ solid solutions on different scale lengths. The primary goal of this investigation is to shed light on the relations between the short-range and the average structure of these materials *via* an analysis of disorder on the mesocopic scale. Real-space (pair distribution function) and reciprocal-space (Rietveld refinement and microstructure probing) analysis of X-ray powder diffraction data and electron spin resonance (ESR) investigations were carried out following this approach. On the local scale, Gd- and Ce-rich *droplets* (*i.e.* small regions a few ångströms wide) form, exhibiting either a distorted fluorite (CeO_2_) or a C-type (Gd_2_O_3_) structure in the whole compositional range. These droplets can then form C-type nanodomains which, for Gd concentrations *x*_Gd_ ≤ 0.25, are embedded in the fluorite matrix. At the site percolation threshold *p*_C_ for a cubic lattice (*x*_Gd_ = *p*_C_ ≃ 0.311), C-type nanodomains percolate inside each crystallite and a structural phase transformation is observed. When this occurs, the peak-to-peak ESR line width Δ*H*_pp_ shows a step-like behaviour, which can be associated with the increase in Gd–Gd dipolar interactions. A general crystallographic rationale is presented to explain the fluorite-to-C-type phase transformation. The approach shown here could be adopted more generally in the analysis of disorder in other highly doped materials.

## Abstract 2

Pair distribution function analysis up to tens of nanometres allows probing of the structural changes in Ce_1−*x*_Gd_*x*_O_2−*x*/2_ solid solutions at varying gadolinium concentrations. Dopant ions and oxygen vacancies form extended Gd_2_O_3_-like clusters (droplets) and nanodomains which, on increasing the Gd concentration, percolate and cause a long-range phase transformation. A general crystallographic rationale is presented which could be adopted to describe phase transformations in highly doped materials.

## 1. Introduction   

The outstanding physical properties of many families of functional oxides are not typical of the pure materials but they emerge when they are suitably doped. This can be considered as an advantage since it allows the fine tuning of their properties. Typically, heavy chemical doping introduces significant disorder in the structure of pristine compounds. In these cases the resulting structure has to be carefully characterized to acquire an exhaustive picture of the physical behaviour.

Structural investigations focus on the modifications induced by doping, either on the average length scale or on the very short length scale, *i.e.* relaxations around dopant ions studied, for example, by the extended X-ray absorption fine structure (EXAFS) technique. Unfortunately, very little attention is usually given to the experimental determination of structural disorder in the so-called mesoscopic range, which defines the boundary between local and long-range structure with correlation domains as large as tens of nanometres.

Cerium oxide is doped with trivalent ions like gadolinium to induce high ionic conductivity (σ_i_); this makes Gd-doped ceria compounds (Ce_1−*x*_Gd_*x*_O_2−*x*/2_) suitable conducting electrolyte candidates to be used in electrochemical cells at intermediate temperatures (800–1000 K) (Steele, 1997 ▸; Goodenough, 2003 ▸; Inaba, 1996 ▸; Zhang *et al.*, 2004 ▸). The Gd doping fraction *x* in Ce_1−*x*_Gd_*x*_O_2−*x*/2_ will be hereinafter denoted *x*_Gd_. Oxygen vacancies are introduced when Gd substitutes for Ce (half vacancy for each doping Gd) (Kilner, 2008 ▸; Scavini & Coduri, 2013 ▸). In these materials, σ_i_ is obtained by O diffusion *via* a vacancy mechanism, which implies microscopic O diffusion from one site to an empty one in its neighbourhood. The defect clustering architecture on different length scales could also influence the diffusion path and should not be neglected.

A clear indication of the effect of disorder on the physical properties of Ce_1−*x*_Gd_*x*_O_2−*x*/2_ comes from the bell-shaped curve of σ_i_ as a function of *x*_Gd_ (Steele, 1997 ▸; Zhang *et al.*, 2004 ▸; Tianshu, 2002 ▸). In particular, σ_i_ (i) increases with *x*_Gd_ up to a critical value (*x*_Gd_ ≃ 0.10) where a broad maximum appears, and (ii) decreases when *x*_Gd_ is increased above ∼0.2 (Zhang *et al.*, 2004 ▸; Scavini & Coduri, 2013 ▸; Tianshu, 2002 ▸). It should be noted that only 5% of the O sites are vacant for *x*_Gd_ = 0.20 and no long-range structural modifications (*i.e.* phase transitions) are detected around this composition value (Grover & Tyagi, 2004 ▸; Zha *et al.*, 2003 ▸; Scavini *et al.*, 2012 ▸; Artini *et al.*, 2012 ▸) through the entire temperature range up to 1073 K (Artini *et al.*, 2014 ▸).

In particular, the structural modifications induced by doping have been explored using local probes such as EXAFS (Yamazaki, 2000 ▸, 2002 ▸; Ohashi, 1998 ▸; Deguchi *et al.*, 2005 ▸), Raman spectroscopy (Banerji *et al.*, 2009 ▸), techniques related to electron microscopy such as high-resolution transmission electron microscopy (HRTEM), electron energy-loss spectroscopy (EELS), selected-area electron diffraction (SAED) (Ye *et al.*, 2009 ▸; Ou *et al.*, 2008 ▸), and atomistic simulations (Burbano *et al.*, 2014 ▸; Butler *et al.*, 1983 ▸; Dholabhai *et al.*, 2012 ▸; Hayashi, 2000 ▸; Inaba, 1999 ▸; Li *et al.*, 2011 ▸; Minervini, 1999 ▸; Wang *et al.*, 2011 ▸; Ye *et al.*, 2008 ▸).

The doping evolution of σ_i_ has been attributed to several effects such as the formation of complex defects, O vacancy ordering, repulsion between vacancies and phase separation on the nanoscale, *i.e.* mechanisms which should induce the trapping of the O vacancies and then reduce the average ionic mobility (Burbano *et al.*, 2012 ▸; Dholabhai *et al.*, 2012 ▸; Inaba, 1999 ▸; Kilner, 2008 ▸; Minervini, 1999 ▸, Steele, 1997 ▸; Tianshu, 2002 ▸).

In our opinion, the presence of complex and spatially extended defects urges a structural study on the mesoscopic scale, *i.e.* the nature and extent of mesoscopic symmetry and compositional fluctuations in Ce_1−*x*_Gd_*x*_O_2−*x*/2_ solid solutions as a function of *x*_Gd_.

Pair distribution function (PDF) analysis from total scattering measurements can be considered as an appropriate technique since it allows studying the structure in terms of the actual interatomic distances (*r*), instead of the average structural information obtained using conventional diffraction methods. In this way, it is in principle possible to observe any deviation from the average structure within the coherence length of a crystallite.

Recently, we performed PDF analysis on the Gd-doped ceria system (Scavini *et al.*, 2012 ▸; Allieta *et al.*, 2011 ▸) and on other dopants (Coduri, Scavini *et al.*, 2012 ▸; Coduri, Brunelli *et al.*, 2012 ▸; Coduri, Scavini, Brunelli & Masala, 2013 ▸; Coduri, Scavini, Brunelli, Allieta & Ferrero, 2013 ▸; Coduri *et al.*, 2014 ▸) as a function of doping concentration. Whilst the compositional evolution of the atomic displacement parameters probed by Rietveld analysis revealed the presence of a large amount of doping-induced disorder, PDF analysis indicated that the disorder spans well above the first coordination shells around the dopant ions. The local scale of doped ceria samples can be pictured as the coexistence of dopant- and Ce-rich *droplets*, *i.e.* small regions (a few ångstroms wide) with either a distorted fluorite (CeO_2_) or C-type (dopant oxide) structure, respectively (Scavini *et al.*, 2012 ▸). The relative proportion of the two regions depends on the system stoichiometry. A discussion of our experimental results with respect to other findings in the existing literature can be found in Scavini *et al.* (2012 ▸).

In addition, a recent PDF investigation of Y-doped ceria (Coduri, Scavini, Brunelli, Allieta & Ferrero, 2013 ▸) showed that, in intermediate dopant compositions (0.25 ≤ *x* < 0.50), the dopant-rich droplets average C-type *domains* a few nanometres wide. This induces evident modifications in the microstructure, since the spread of the nanodomains leads to the formation of antiphase boundaries (APB).

In view of their superior performance as electrolytes compared with other dopants, we propose to deepen the structural investigation of Gd-doped samples reported by Scavini *et al.* (2012 ▸), filling the compositional gap (0.25 < *x*_Gd_ < 0.50) by considering intermediate compositions and extending the real-space analysis to a range of tens of nanometres. For a better understanding of the present work, we will recall some results reported by Scavini *et al.* (2012 ▸).

The present structural analysis is accompanied by electron spin resonance (ESR), which acts as a local magnetic probe of Gd ions, with the aim of examining the evolution of dipolar interactions upon doping.

Finally, we will provide a general crystallographic argument to elucidate the fluorite to C-type phase transformation mechanism, based on the percolation of hierarchical defect structures.

We believe that the approach shown here can be generally followed for the analysis of disorder in other highly doped materials. This may be of fundamental importance to match structural pieces of information at different length scales by enlightening the structure–physical properties relationship.

## 2. Experimental   

### 2.1. Sample preparation   

Micro-crystalline Ce_1−*x*_Gd_*x*_O_2−*x*/2_ samples with Gd concentrations *x*_Gd_ spanning the whole solid solution range were prepared by applying the Pechini sol–gel method (Pechini, 1967 ▸; Rezaei *et al.*, 2009 ▸). Ce nitrate Ce(NO_3_)_3_·6H_2_O (Aldrich, ≥99%) and Gd nitrate Gd(NO_3_)_3_·6H_2_O (Aldrich, 99.9%) were used as precursors in stoichiometric ratio, while ethylene glycol (Aldrich, ≥99%) and citric acid (Aldrich, 99%) were added as polymerization agents for the process. The resulting gel was burned in an ashing furnace (Nabertherm), heated at a rate of about 3 K min^−1^ up to 773 K, and then kept stable at this temperature for 3 h. The powder produced was then pressed into pellets and fired at 1173 K for 72 h in air.

### 2.2. Data collection   

X-ray powder diffraction (XRPD) measurements were performed on all samples, as well as on CeO_2_ (Aldrich, ≥99.0%) and Gd_2_O_3_ (Aldrich, 99.9%), using the high-resolution diffractometer on the ID31 beamline of the ESRF (the European Synchrotron, Grenoble, France; Fitch, 2004 ▸).

The samples were loaded into glass capillaries (1.0 mm diameter), mounted on the diffractometer axis and spun during measurements in order to promote powder randomization. The samples were cooled to *T* = 90 K using a liquid nitrogen gas blower (Oxford Cryosystems) mounted coaxially; the set point of *T* = 90 K was selected in order to minimize the atomic thermal vibrations, which cause broadening of the PDF peaks in real space.

Data were collected during two different experiments. For *x*_Gd_ = 0.313, 0.344, 0.375 and 0.438, an X-ray wavelength λ = 0.35412 (1) Å was used in the angular range 0 < 2θ < 120°, covering a range of the wavevector *Q* (= 4π sinθ/λ) up to *Q*_max_ ≃ 29.4 Å^−1^, while for the compositions *x*_Gd_ = 0, 0.125, 0.25, 0.50, 0.75, 0.875 and 1, data were collected at λ = 0.30975 (1) Å in the range 0 < 2θ < 100° with *Q*_max_ ≃ 31 Å^−1^ (see Scavini *et al.*, 2012 ▸).

In all cases, the counting time at higher angles was much longer, in order to increase the statistical significance of the data.

ESR measurements were carried out on the same samples and, in addition, on samples with *x*_Gd_ = 0.05 and 0.20, using a Bruker ELEXSYS spectrometer equipped with an ER4102ST standard rectangular cavity at *X* band (9.4 GHz) frequency and at room temperature. The powdered samples were placed in a quartz tube and the derivative d*P*/d*H* of the absorbed power *P* was recorded as a function of the static magnetic field *H*.

### 2.3. XRPD data analysis   

The XRPD patterns were analysed *via* the Rietveld method as implemented in the *GSAS* software suite of programs (Larson & Dreele, 2004 ▸), which feature the graphical user interface *EXPGUI* (Toby, 2001 ▸). Deviations from the long-range structure were investigated by means of the PDF method. For this purpose, we used the so-called reduced PDF, *G*(*r*), which can be obtained *via* the Fourier sine transform of the experimental total scattering function *S*(*Q*) 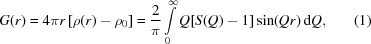
where ρ(*r*) is the atomic number density function and indicates the probability of finding an atom at a distance *r* from another atom, while ρ_0_ is the average number density.

The *G*(*r*) curves corresponding to the experimental data were computed using the program *PDFGetX2* (Qiu *et al.*, 2004 ▸). Only data up to *Q*_max_ ≃ 28 Å^−1^ were used to obtain the experimental *G*(*r*) curves because of the insufficient signal-to-noise ratio at higher *Q* values. After background subtraction, the data were corrected for sample self-absorption and for multiple and Compton scattering. The *G*(*r*) analysis was carried out *via* the so called ‘real-space Rietveld’ method (Egami & Billinge, 2003 ▸) featured in the program *PDFGui* (Farrow *et al.*, 2007 ▸). To avoid misunderstanding, we will use ‘Rietveld’ to denote the Rietveld data analysis in *Q* space and ‘real-space Rietveld’ to denote the Rietveld-like data analysis in real space.

The extremely narrow instrumental resolution function of ID31 (Fitch, 2004 ▸) leads to small Gaussian damping envelopes in real space (Farrow *et al.*, 2007 ▸). This allows the calculation of *G*(*r*) functions over several hundreds of ångströms, implying that an investigation of the local structure in real space is feasible over a large interatomic range.

The degree of accuracy of the analysis is defined by the residual factor 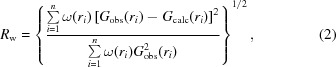
where ω(*r*_*i*_) = 1/σ^2^(*r*_*i*_) and σ(*r_i_*) is the standard deviation at a distance *r*_i_.

The low *r* range of the *G*(*r*) curves (∼2 < *r* < ∼6 Å) was also investigated using the so-called direct analysis method, as described by Coduri, Brunelli *et al.* (2012 ▸). For this purpose, the *G*(*r*) peaks were fitted using Gaussian functions after subtraction of the linearly fitted baseline.

## 3. Results and discussion   

### 3.1. XRPD   

#### 3.1.1. Fluorite and C-type crystal structures   

**Figure 1:**
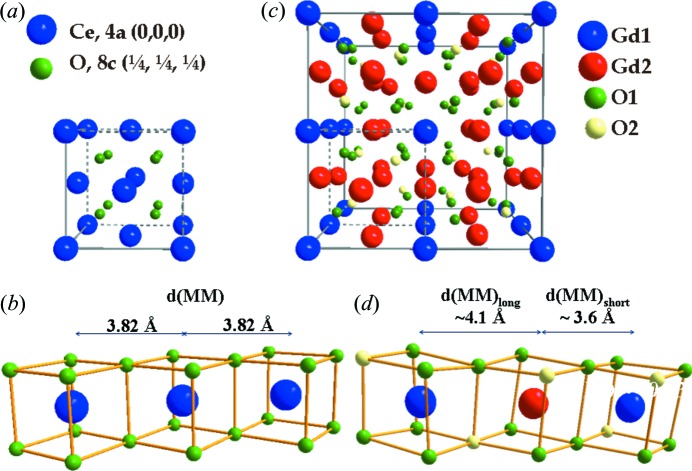
(*a*) The unit cell and (*b*) the cation chemical environment of CeO_2_. (*c*) The unit cell and (*d*) the cation chemical environment of Gd_2_O_3_. *d*(*MM*) is the unique Ce–Ce NN pair distance in CeO_2_, while in Gd_2_O_3_ two different NN Gd–Gd distances are present, *d*(*MM*)_short_ and *d*(*MM*)_long_.

Let us start by describing the reference crystal structures. Pure ceria exhibits the fluorite structure in space group 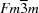 (No. 225). The Ce atom occupies a site *M*: 4*a*, (0, 0, 0). The O atom is located in a site O: 8*c*, (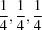). Ce is eight-fold coordinated (see Fig. 1 ▸ *a*). All the Ce–Ce next-nearest neighbour (NNN) distances are identical in the fluorite structure (see Fig. 1 ▸ *b*). In Ce_1−*x*_Gd_*x*_O_2−*x*/2_ fluorite solid solutions, O vacancies are introduced into the O site and the occupation factor (o.f.) of the latter becomes o.f.(O) = 1 − *x*_Gd_/4.

Gd_2_O_3_ exhibits the so-called C-type structure in space group 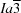 (No. 206). Two Gd sites are present, *M*1: 8*b* (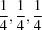) and *M*2: 24*d* (*x*, 0, 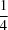) with *x*(*M*2) ≃ −0.03, and one O anionic site, O1: 48*e*, (*x*, *y*, *z*) with *x*(O1) ≃ 0.39, *y*(O1) ≃ 0.15, *z*(O1) ≃ 0.38 (Scavini *et al.*, 2012 ▸). In Fig. 1 ▸(*c*) the Gd_2_O_3_ unit cell is illustrated; the unit-cell origin is shifted in order to highlight the close relationship with the fluorite structure. All the Gd ions have six-fold coordination. ‘Short’ and ‘long’ NNN *M*—*M* distances [*d*(*MM*)_short_ and *d*(*MM*)_long_, respectively] are present as a consequence of the non-zero *x* coordinate of the Gd2 site (see Fig. 1 ▸ *d*). In particular, ‘long’ NNN interatomic distances separate cations when an O vacancy occurs on the edge connecting their coordination polyhedra, while ‘short’ NNN distances are interposed when two full O sites form the same edge (see Fig. 1 ▸ *d*). In C-type solid solutions, another O site, O2 [16*c*, (*x*, *x*, *x*], where *x*(O2) ≃ 0.389, becomes partially filled, with o.f.(O2) = 1 − *x*_Gd_.

Although there is no group–subgroup relationship between the fluorite and C-type phases, they are closely related to each other. With respect to fluorite, the C-type unit-cell axes double, as a consequence of the O vacancy ordering and the related atomic position displacements. Starting from a 2 × 2 × 2 replica of the fluorite cell, it is possible to obtain the C-type structure by shifting the cell origin by (0, 0, −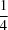) and the atomic positions by suitable displacements Δ. The positional degrees of freedom in the C-type phase can be rewritten as *x*(*M*2) = 0 + Δ*x*(*M*2), *x*(O1) = 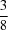 + Δ*x*(O1), *y*(O1) = 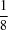 + Δ*y*(O1), *z*(O1) = 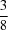 + Δ*z*(O1), *x*(O2) = 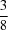 + Δ*x*(O2) and *z*(O2) = 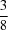 + Δ*x*(O2). If all the Δ*x*, Δ*y*, Δ*z* values are fixed at zero and o.f.(O2) = 1, the resulting structure is that of fluorite. The relationship between the two structures is reported schematically in Table S1 of the supporting information.

This relationship is reflected in the XRPD pattern of the C-type phase, with the emergence of additional peaks (hereinafter denoted ‘superstructure’ peaks) besides the fluorite characteristic peaks (hereinafter denoted ‘structure peaks’).

#### 3.1.2. Reciprocal space analysis   

Rietveld refinements and related optimized parameters referring to the samples with *x*_Gd_ = 0, 0.125, 0.25, 0.50, 0.75, 0.875 and 1 have already been shown by Scavini *et al.* (2012 ▸). The analogous parameter values for samples *x*_Gd_ = 0.313, 0.344, 0.375 and 0.438 are reported in Table S2, whereas the respective Rietveld refinements are shown in Fig. S1. The structure turns from fluorite to C-type for *x*_Gd_ = 0.313, and superstructure peaks appear and are broader than the structure peaks.

**Figure 2:**
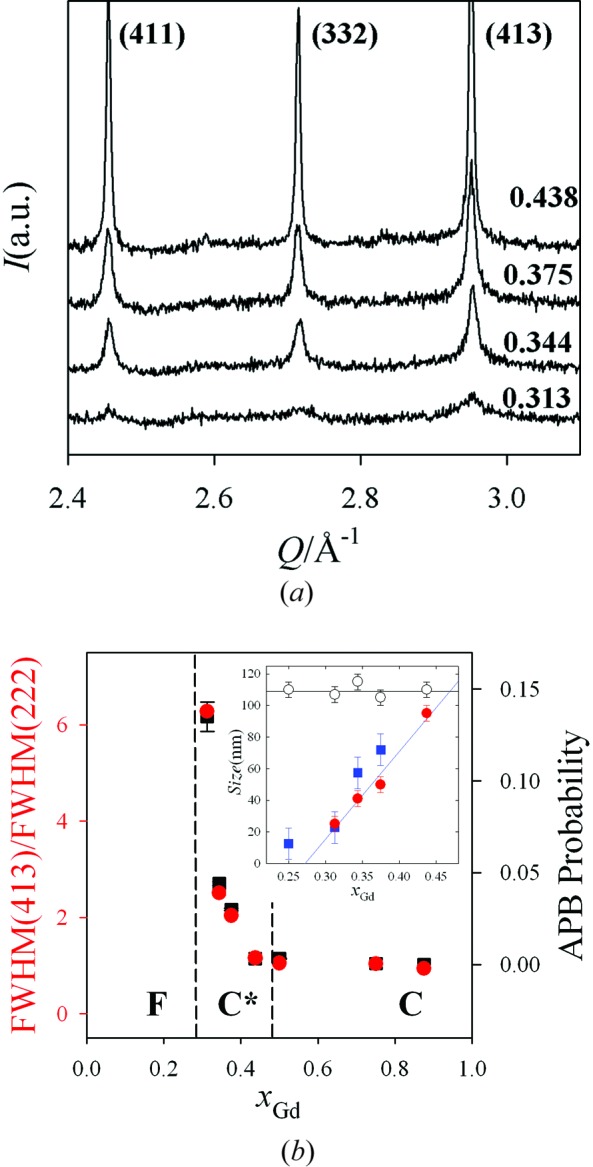
(*a*) A portion of the experimental patterns of Ce_1−*x*_Gd_*x*_O_2−*x*/2_ solid solutions in the C* zone. (*b*) FWHM(413)/FWHM(222) and APB concentration in the C* and C zones. (Inset) Size parameters referring to superstructure (red circles) and structure (empty circles) peaks as obtained by Williamson–Hall analysis, and by PDF analysis (blue squares).

Fig. 2 ▸(*a*) depicts a small portion of the experimental patterns, including the most intense superstructure peaks, *i.e.* (411), (332) and (413), consistent with the C-type metrics for samples in the range 0.25 < *x*_Gd_ < 0.50. These reflections strengthen and sharpen with increasing *x*_Gd_. The ratio between the full widths at half maximum (FWHM) of the (413) superstructure and the (222) structure peaks is shown in Fig. 2 ▸(*b*) (red circles). It is worthwhile noticing that FWHM(413) is about six times FWHM(222) for *x*_Gd_ = 0.313.

For *x*_Gd_ > 0.313, the superstructure peaks strengthen and the FWHM(413)/FWHM(222) ratio decreases, approaching 1 for *x*_Gd_ ≥ 0.50. Hereinafter, as proposed by Coduri, Scavini, Brunelli, Allieta & Ferrero (2013 ▸) for Y-doped ceria, we will call C* and C the crystal structures in the ranges 0.313 ≤ *x*_Gd_ < 0.50 and 0.50 ≤ *x*_Gd_ ≤ 1.00, respectively. The differences between these two compositional zones are shown and discussed below. However, it should be noted that they both belong to the C-type structure and no intermediate phase transition occurs.

It should be recalled that diffraction peaks broaden as a result of effects limiting the coherence of the lattice. Typical examples are the so-called extended defects, such as dislocations, material strains, APBs and finite crystallite sizes. In this regard, irrespective of the defect type, it is possible to extract a reference size parameter linked to the spatial extent of the defects. Since in the C* region both structure and superstructure peaks show different broadenings, in the inset of Fig. 2 ▸(*b*) we report the respective size parameters, as extracted using the Williamson–Hall approximation (Williamson & Hall, 1953 ▸). While the size derived from the structure reflections (hollow circles) stays almost constant for the different samples, the size determined from the superstructure reflections (solid red circles) increases almost linearly with *x*_Gd_ and approaches the size extracted from the structure reflections at *x*_Gd_ = 0.428.

On the other hand, since extended defects such as dislocations and APBs (Scardi & Leoni, 2005 ▸; Coduri, Scavini, Brunelli, Allieta & Ferrero, 2013 ▸) can also cause (*hkl*)-dependent peak broadening, we adopted whole powder pattern modelling (WPPM), which allows discrimination between the different broadening sources on the basis of their (*hkl*) broadening dependencies. Patterns in the 0.313 ≤ *x*_Gd_ ≤ 0.438 range were suitably fitted considering only the presence of randomly distributed APBs. In Fig. 2 ▸(*b*), the APB probability is plotted as a function of *x*_Gd_ (black squares), while the best fits are shown in Fig. S2.

From the Rietveld analysis it appears that, with increasing *x*_Gd_, all Δ values move gradually from 0 to the values found for pure Gd_2_O_3_. In particular, the *x*(*M*2) [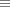 Δ*x*(*M*2)] parameter can be considered as a fingerprint of O vacancy concentration and ordering. Actually, when an O vacancy forms, the *M*2 ion position is shifted along one crystalline axis and two different NNN *M*–*M* distances are present, as shown in Fig. 1 ▸(*d*) for the case of pure Gd_2_O_3_.

**Figure 3:**
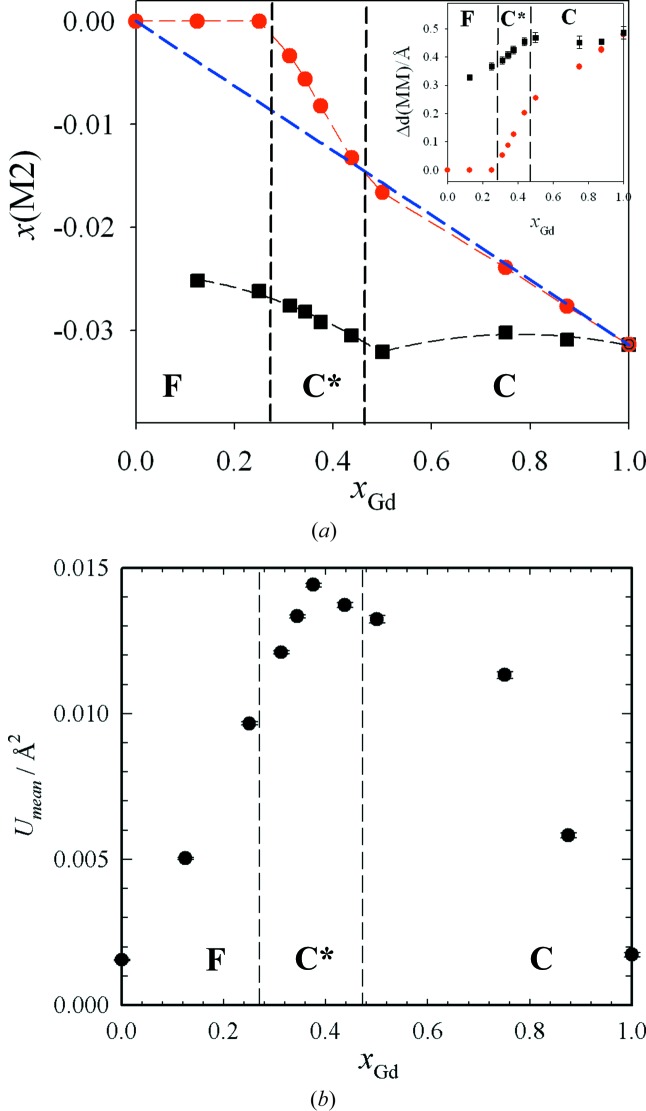
(*a*) *x*(*M*2) values *versus* the composition *x*_Gd_ as obtained by reciprocal-space (red circles) and real-space (black squares) analysis. (Inset) The difference between the ‘long’ and ‘short’ *M*–*M* distances, Δ*d*(*MM*), as obtained by reciprocal-space (red circles) and real-space (black squares) analysis. (*b*) Average atomic mean-square displacement *U*_mean_ plotted as a function of *x*_Gd_.

Fig. 3 ▸(*a*) reports the *x*(*M*2) values (red circles) as a function of *x*_Gd_. Three different linear trends are apparent. In the fluorite zone, *x*(*M*2) is fixed to zero, whereas in the C* and C zones *x*(*M*2) decreases linearly *versus* increasing Gd concentration, but with two different slopes. In the inset, the difference between ‘long’ and ‘short’ *M*–*M* distances Δ*d*(*MM*) [= *d*(*MM*)_long_ − *d*(*MM*)_short_] is shown as a function of composition (red circles). In the fluorite region, Δ*d*(*MM*) 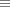 0 because there is only one NNN *M*–*M* distance. Δ*d*(*MM*) increases monotonically with further increases in the Gd concentration and reaches a value as large as ∼0.5 Å for Gd_2_O_3_.

In Fig. 3 ▸(*b*), the average atomic mean-square displacement (msd) parameters are plotted as a function of composition. Since all data were collected at the same temperature, the msd parameters can be considered as fingerprints of disorder. Starting from CeO_2_, the msd values increase when varying *x*_Gd_, reaching a maximum for *x*_Gd_ ≃ 0.375, and then decrease monotonically up to *x*_Gd_ = 1 (*i.e.* pure Gd_2_O_3_). In the C* zone, the msd values are about one order of magnitude larger than in the pure materials, suggesting the presence of very high disorder in the solid solutions.

However, Rietveld analysis is not the most appropriate method to supply a microscopic description of disorder. As a consequence, we switched over to real-space analysis, starting from the first interatomic distances and then approaching spatial domains encompassing tens of nanometres.

#### 3.1.3. Real-space analysis   

**Figure 4:**
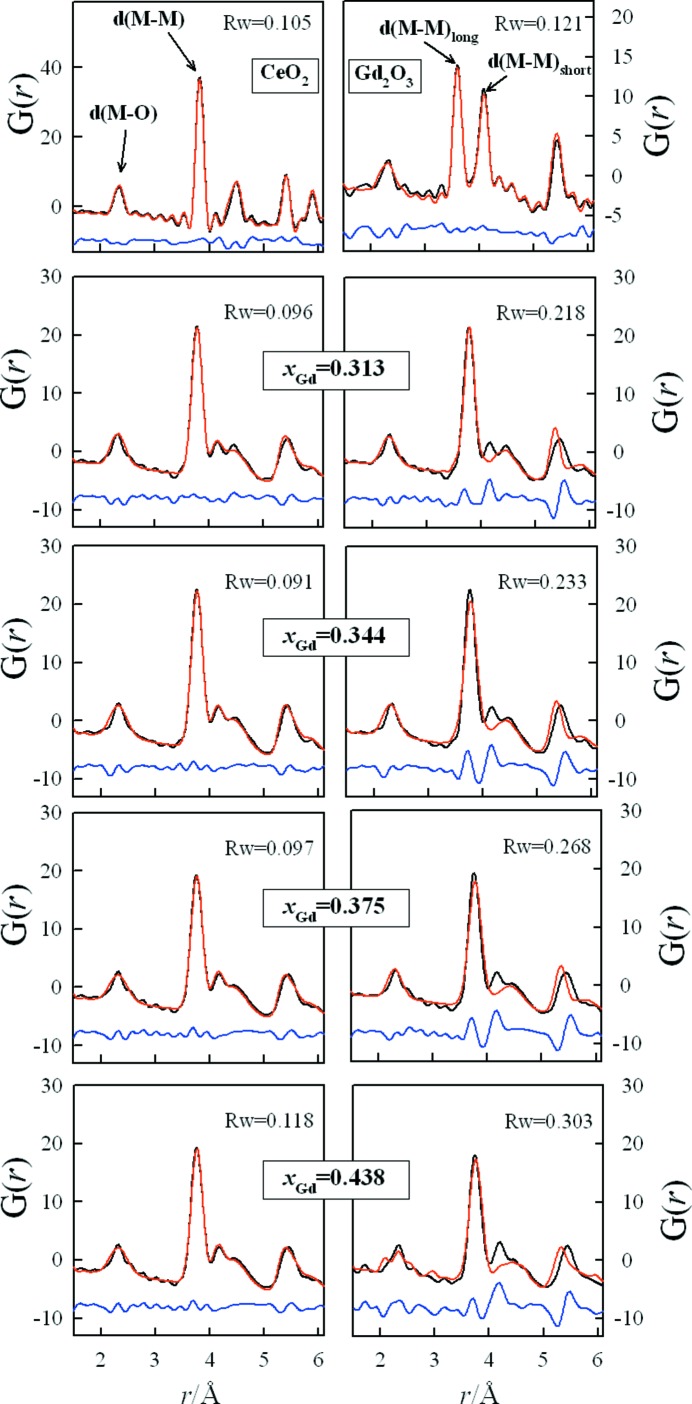
Real-space Rietveld refinements of *G*(*r*) pertinent to the samples *x*_Gd_ = 0, 0.313, 0.344, 0.375, 0.438, 1 in the 1.5 < *r* < 6 Å range. Measured (black lines) and calculated (red lines) profiles are shown, along with residuals (blue lines). For solid solutions, both biphasic (left) and monophasic (right) models are displayed, while for the CeO_2_ and Gd_2_O_3_ samples only the monophasic model fits are shown.

The experimental PDFs for the C* region are shown as black dots in Fig. 4 ▸, together with those of the pure oxides. The first peak in the PDF of all the samples corresponds to the *M*–O NN distance *d*(*M*–O), the second peak is due to the unique *M*–*M* NNN distance *d*(*MM*) in CeO_2_ and the shortest *M*–*M* distance *d*(*MM*)_short_ in Gd_2_O_3_, and the third one is the signature of the longest *M*–*M* NNN distance in Gd_2_O_3_, *i.e.* *d*(*MM*)_long_.

The PDF analysis of both fluorite and C regions of the CeO_2_–Gd_2_O_3_ solutions was detailed earlier by Scavini *et al.* (2012 ▸). It was shown that, in the very short range, EXAFS and PDF measurements yield similar results. In particular, the decrease in *d*(*M*–O) in the fluorite zone agrees well with the element-sensitive EXAFS results of Ohashi (1998 ▸). It is to be noted that the latter were weighted on the Gd/Ce concentration for the sake of comparison [see Fig. 4*b* of Scavini *et al.* (2012 ▸)].

For the metal–metal distances, direct analysis of samples with an average fluorite structure provided evidence of C-type ordering, as revealed by the appearance of the peak corresponding to *d*(*MM*)_long_. The trend of this distance against composition was not consistent with that expected from the average structure and suggested that the additional PDF peak in the fluorite structure should be assigned to the longer *M*–*M* pair distance involving the dopant, typical of C-type ordering. This finding was also supported by an anomalous differential PDF study (Allieta *et al.*, 2011 ▸) and by EXAFS measurements at the Ce *K* and Gd *K* edges (Dholabhai *et al.*, 2012 ▸), which provided evidence of longer Gd–Gd NNN distances compared with the Ce–Ce and Ce–Gd ones in the whole investigated compositional range (*x*_Gd_ ≤ 0.30).

The Δ*d*(*MM*) values obtained by PDF analysis for all the samples by subtracting the *d*(*MM*)_long_ distances from the *d*(*MM*)_short_ distances are reported in the inset of Fig. 3 ▸(*a*) (black squares). Δ*d*(*MM*) increases almost linearly *versus x*_Gd_ in the fluorite and C* regions, while it remains constant in the C region.

Finally, the local scale can be pictured by means of the real-space Rietveld approach applying a biphasic model, which implies the coexistence of CeO_2_-like and Gd_2_O_3_-like droplets. The same model applied to fluorite and C regions by Scavini *et al.* (2012 ▸) is now extended to the samples in the C* region. The biphasic model best fits (left-hand side), together with those for the average structure (right-hand side), are reported in Fig. 4 ▸ as red lines. The results for the pure oxides are also plotted for reference, using their average structures.

The model fitting parameters (Table S3) and details of the models used are reported in the supporting information, while the *x*(*M*2) values found *via* the biphasic model for the C-type phases are compared with the Rietveld results in Fig. 3 ▸(*a*) (black squares) for all the samples. PDF analysis revealed that C-type droplets are present even in samples with the lowest investigated Gd concentrations: for *x*_Gd_ = 0.125, already *x*(*M*2) = −0.025, *i.e.* ∼80% with respect to Gd_2_O_3_. *x*(*M*2) decreases monotonically with increasing *x*_Gd_ up to *x*_Gd_ = 0.50, and then it approaches the value characteristic of Gd_2_O_3_.

The above results imply a noticeable O vacancy ordering on the local scale in all the solid solutions, much more extended than is foreseen by the average model.

In order to reconcile the findings at different length scales, we expanded the investigation in the *r* space using real-space Rietveld analysis in different interatomic ranges.

First, the biphasic model was applied to wider *r* ranges (up to ∼20 Å) in the fluorite and C* zones using spatial fitting ranges of about 5 Å, while keeping the C-type phase fraction fixed (as determined in the short range) and allowing only variation in cell constants, msd parameters and *x*(*M*2).

**Figure 5:**
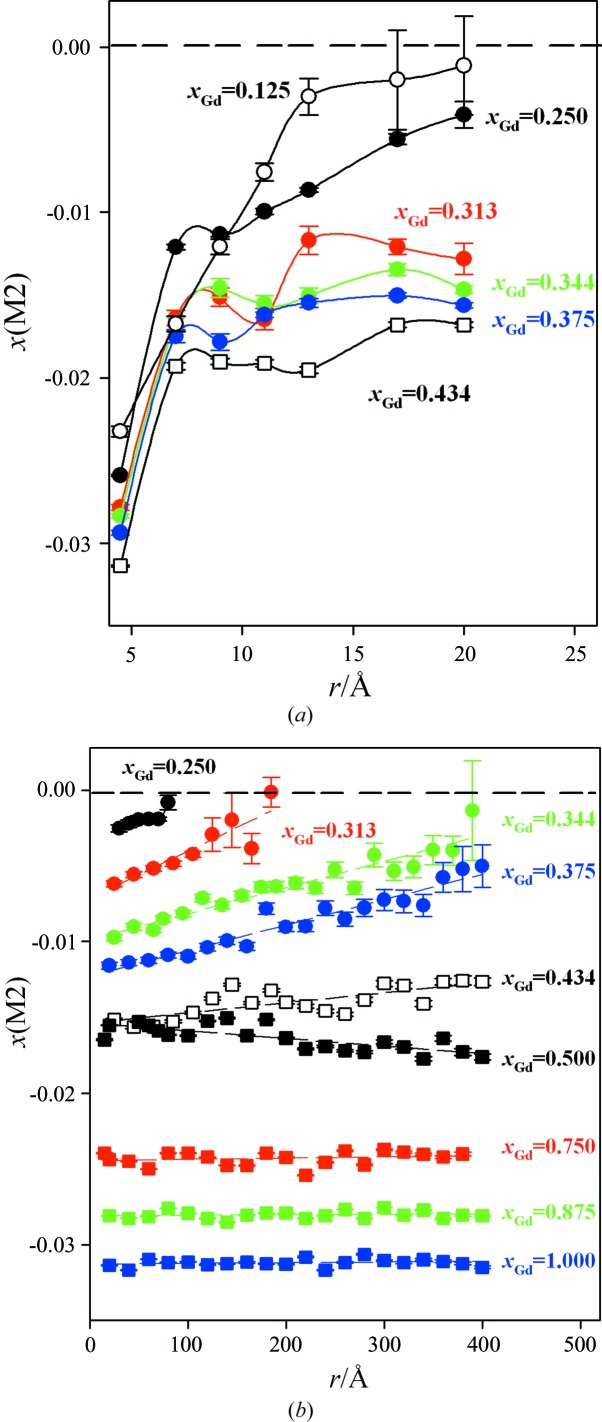
(*a*) A plot of the *x*(*M*2) trend as a function of *r* using the biphasic model. (*b*) The same as part (*a*) but using the C-type model. In both cases, the *r* values are the ‘centroids’, *i.e.* the mean values of the different *r* ranges considered in the refinement.

The behaviour of *x*(*M*2) for samples in the fluorite and C* zones is reported in Fig. 5 ▸(*a*) as a function of *r*. Despite the data dispersion, it is possible to distinguish some trends. For all the samples considered, *x*(*M*2) increases rapidly at increasing *r* up to *r* = 10–15 Å, and then its gradient decreases. The steep increase in *x*(*M*2) *versus r* suggests that the correlation length of the CeO_2_ and Gd_2_O_3_ droplets is very short, as pointed out by Scavini *et al.* (2012 ▸). For *x*_Gd_ = 0.125, *x*(*M*2) approaches zero already at *r* ≃ 15 Å. Conversely, *x*(*M*2) ≠ 0 for all the other samples even at larger *r* values.

To expand the PDF analysis to an *r* limit of 400 Å, we adopted a box-car refinement approach using *r* steps as large as 20 Å. The experimental *G*(*r*) patterns were fitted only by the C-type structural model, since extending the biphasic model to larger *r* values would introduce high correlations between the parameters. It should be noted that, applying suitable constraints in the biphasic model, the two models lead to equivalent results, but it must be borne in mind that in the C-type model parameters like *x*(*M*2) are averaged over the whole *G*(*r*) function, while in the biphasic model the same parameters are averaged solely over the C-type fraction.

To avoid correlations between parameters in the C-type model, O positions and occupancies were kept fixed to the Rietveld results (see Table S2), varying only one scale factor, one cell constant, two msd values and *x*(*M*2) in the subsequent optimization.

A Nyquist grid was utilized to avoid oversampling (Farrow *et al.*, 2011 ▸). The refined *x*(*M*2) *versus r* curves are reported in Fig. 5 ▸(*b*).

Starting from the fluorite solid solutions, a non-zero value of *x*(*M*2) is obtained up to *r* ≃ 8 nm for the *x*_Gd_ = 0.25 sample. In the 0.25 ≤ *x*_Gd_ ≤ 0.438 range, a common feature for all samples is a positive slope in the *x*(*M*2) *versus r* plot, which decreases with increasing *x*_Gd_. In this compositional range, one can calculate the *r* intercepts as a function of *x*_Gd_ *via* linear regression of the *x*(*M*2) data (dashed lines). These data are plotted in the inset of Fig. 2 ▸(*b*) (blue squares) and match quite well the size parameter obtained *via* the Williamson–Hall method from the broadening of the superstructure peaks. In the C zone, *x*(*M*2) stays constant with varying *r*, in agreement with the values obtained by Rietveld refinement. This agreement, found on an absolute scale, corroborates the correctness of our approach.

### 3.2. ESR   

**Figure 6:**
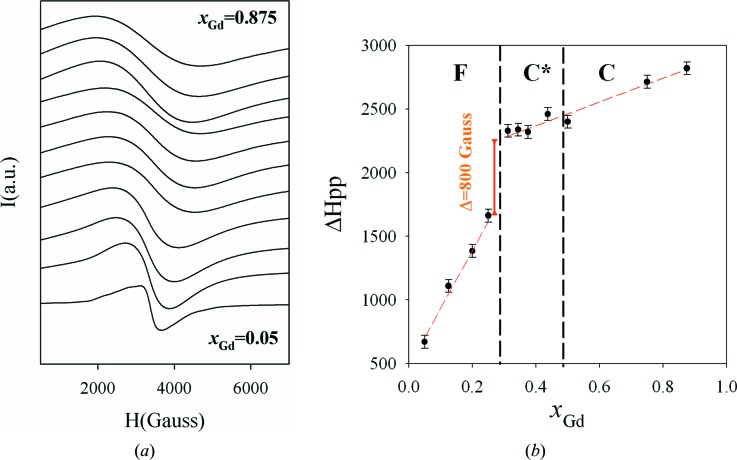
(*a*) ESR spectra at room temperature. Starting from the bottom, *x*_Gd_ = 0.05, 0.125, 0.20, 0.25, 0.313, 0.344, 0.375, 0.438, 0.50, 0.75, 0.875, 1. (*b*) The peak-to-peak line-width (Δ*H*_pp_) profile for the same samples.

It is well known that Gd_2_O_3_ can be deemed to be a prototype paramagnetic system where a single asymmetric broad resonance line is determined by the wide distribution of Gd–Gd dipolar fields (Tobia *et al.*, 2014 ▸). On the other hand, CeO_2_ is an ESR-silent compound as its signal cannot be observed due to its almost negligible paramagnetism. Therefore, we decided to analyse all the above-mentioned solid solutions by means of ESR spectroscopy, aiming to investigate the interactions between the Gd ions. Fig. 6 ▸(*a*) shows the ESR spectra collected at room temperature for all the samples.

For *x*_Gd_ = 0.05, the ESR spectrum displays several broad lines belonging to Gd^3+^ transitions. This spectrum resembles the spectra obtained from dilute solid solutions (de Biasi & Grillo, 2005 ▸). For *x*_Gd_ ≥ 0.125, the transition lines are too broad to be fully resolved and a single broad resonance line (*g*_eff_ ≃ 2) can be observed in all spectra. The line width increases with increasing Gd concentration. The fitting performed on Ce_1−*x*_Gd_*x*_O_2−*x*/2_ spectra to extract quantitative parameters using a single Lorentzian or Gaussian function generally gave poor results, as observed previously (Oliva *et al.*, 2004 ▸). The Dysonian line shape (Joshi & Bhat, 2004 ▸; Allieta *et al.*, 2013 ▸; Oliva *et al.*, 2015 ▸) including more parameters provided a more satisfactory description of the ESR line shapes but only for *x*_Gd_ ≥ 0.375. As a consequence, to show the evolution of the ESR spectra upon doping in the whole compositional range, we decided to extract the peak-to-peak line width (Δ*H*_pp_) by direct inspection of the experimental patterns. The trend of Δ*H*_pp_ against Gd concentration is shown in Fig. 6 ▸(*b*) for all samples.

Δ*H*_pp_ increases rapidly in the fluorite zone with increasing *x*_Gd_. After an abrupt 800 Gauss jump at the boundary between fluorite and C* solid solutions, Δ*H*_pp_(*x*_Gd_) increases linearly again but with a much smaller slope. To analyse the trend below and above the step-like increase in Δ*H*_pp_ for *x*_Gd_ > 0.25, the data were fitted using a linear relation parametrized as follows: Δ*H*_pp_ = Δ*H*_pp_^0^ + *bx*_Gd_, where Δ*H*_pp_^0^ is the intrinsic line width and *b* is a constant. In the intervals 0.05 ≤ *x*_Gd_ ≤ 0.25 and 0.313 ≤ *x*_Gd_ ≤ 0.875 we found Δ*H*_pp_^0^ = 460 G, *b* = 4739, and Δ*H*_pp_^0^ = 2054 G, *b* = 821, respectively.

As reported for dilute solid solutions (de Biasi & Grillo, 2005 ▸), the increase in dipolar broadening in diamagnetic CeO_2_ is described by a relation of the type Δ*H*_pp_ = Δ*H*_pp_^0^ + *c*_1_ *f*_e_, where *f*_e_ is the concentration of substitutional ions and *c*_1_ is a constant which depends mainly on the range of exchange interaction between the paramagnetic ions. Similarly, the observed *x*_Gd_-dependent broadening and the dramatic increase in Δ*H*_pp_ at the fluorite–C* boundary can be associated with the increase in Gd–Gd dipolar interactions at the phase transition. On the other hand, the drop in the *b* parameter in the second regime, *i.e. x*_Gd_ ≥ 0.313, seems to be consistent with a transition from a *first* phase, in which small variations in *x*_Gd_ induce a rapid increase in exchange interactions, to a *second* phase. From the point of view of Gd–Gd interactions, the latter phase seems to be more homogenous, since a single line featuring a smooth variation in dipolar broadening is observed up to *x*_Gd_ = 0.875.

## 4. General discussion   

The real-space PDF analysis shows that, in the very short *r* range (less than ∼1 nm), Ce_1−*x*_Gd_*x*_O_2−*x*/2_ solid solutions can be suitably described using a biphasic model where both CeO_2_ (F) and Gd_2_O_3_ (C) ‘droplets’ coexist and exhibit a continuous structural evolution passing from the fluorite to the C* and C solid solutions. On the other hand, the reciprocal-space (Rietveld) analysis reveals the presence of a structural phase transformation from space group 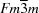 to 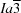. At the same time, the behaviour of Δ*H*_pp_ *versus x*_Gd_ differs in the two phases, exhibiting a step 800 Gauss wide at their boundary.

**Figure 7:**
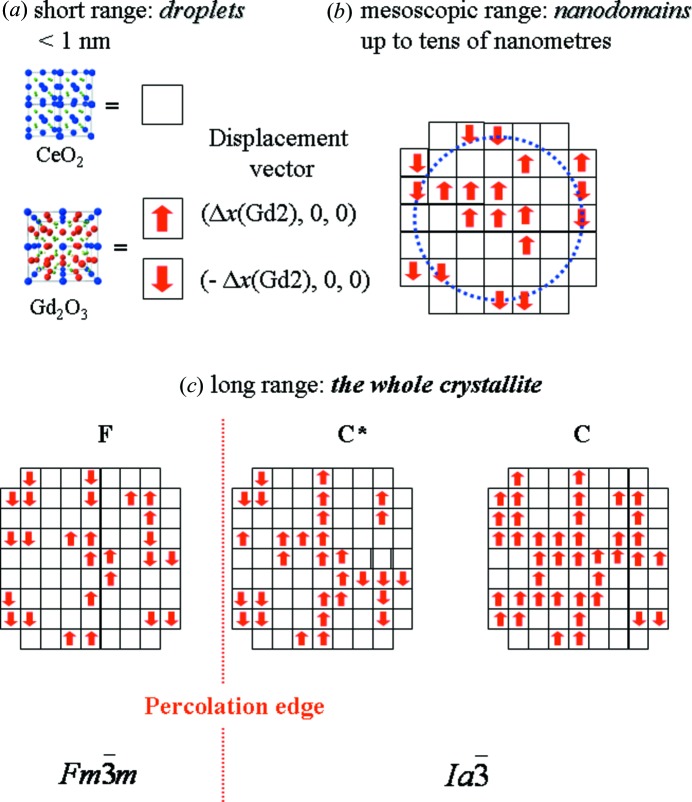
A pictorial representation of the mixing of CeO_2_-like and Gd_2_O_3_-like droplets on different length scales.

In the following, we will use the biphasic model as a suitable starting point for a bottom-up representation of the structure of solid solutions in order to establish a unique consistent framework for the structural findings on different length scales, including the spectroscopic results. The model is illustrated in Fig. 7 ▸.

Droplets (see Figs. 7 ▸ *a* and 7 ▸ *b*) are connected to each other by three kinds of contact, namely F–F, F–C and C–C. F–F are trivial contacts because they only enlarge the droplet correlation length due to the isotropic orientation of the 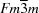 structure. On the other hand, F–C contacts should increase the overall enthalpy content of the system because droplets of different symmetries are involved, in which cations and anions have different equilibrium positions.

Different C–C contacts can exist due to the positional degrees of freedom of atoms *M*2, O1 and O2 in the C-type structure, as pointed out in §3.1.1. To outline the interplay between C–C contacts and droplet symmetry, we introduce in the following the concept of ‘droplet orientations’. A Gd_2_O_3_-like droplet can ideally be created starting from a perfect fluorite structure, *e.g.* by moving the cation sited at (0, 0, 0) along the 〈100〉 direction by a step of components (Δ*x*(Gd2), 0, 0), where Δ*x*(Gd2) stands for the value of Δ*x*(*M*2) in pure Gd_2_O_3_. The remaining metal and oxygen sites are displaced within the correlation length of the droplet according to the C-type structure and the 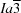 space group operators, creating O vacancies at the O2 sites. In the same way, C-type droplets could also be created *via* different displacement vectors of components: (−Δ*x*(Gd2), 0, 0), (0, Δ*x*(Gd2), 0), (0, −Δ*x*(Gd2), 0), (0, 0, Δ*x*(Gd2)) and (0, 0, −Δ*x*(Gd2)).

According to these displacement vectors, neighbouring C droplets can have either the same or different orientations. In the former case, a droplet is enlarged in terms of correlation length, whereas in the latter case an antiphase surface is produced, giving rise to an increase in the system’s enthalpy. To illustrate this model, Fig. 7 ▸ displays a simplified picture, where only positive and negative displacements in one direction are allowed for sake of clarity.

To estimate the possible evolution of droplet connectivity upon doping, we examine the *r*-dependence of the structural parameter *x*(*M*2) obtained by PDF analysis. For all the compositions investigated, when the biphasic model is applied to *r* ranges, each up to ∼20 Å, *x*(*M*2) increases rapidly with increasing *r* (see Fig. 5 ▸ *a*), suggesting that the correlation length of pure Gd_2_O_3_ and CeO_2_ droplets is very short-range. C–F and/or C–C surfaces with different orientations are thus not uncommon.

For *x*_Gd_ < 0.375, *x*(*M*2) differs from 0 within a few nanometres, even when the average structure is well described by the fluorite model (see Fig. 5 ▸). This means that C-type nanodomains form, which are atomic arrangements in which the probability of finding C droplets with a given displacement vector orientation is larger than in the opposite sense. This is illustrated pictorially in Fig. 7 ▸(*b*).

The behaviour of the *x*(*M*2) parameter *versus x*_Gd_ and *r* suggests that the concentration of C-type domains and/or the degree of order inside them increases when *x*_Gd_ increases. Moreover, for all the samples up to *x*_Gd_ < 0.500 (*i.e.* for fluorite and C* solid solutions), and by increasing *r*, *x*(*M*2) increases smoothly. Conversely, for *x*_Gd_ ≥ 0.500 (*i.e.* for the C solid solutions), *x*(*M*2) stays constant and close to its long-range value up to the largest investigated *r* values.

Recalling that in the 0.25 ≤ *x*_Gd_ < 0.438 range (Fig. 2 ▸ *b*) the intercept of *x*(*M*2) as a function of *r* is in agreement with the evolution of the size parameter, there is clear evidence that the superstructure peak broadening observed in XRPD patterns is closely related to the coherence length of nanodomains as estimated by the PDF technique. Actually, this picture is also consistent with the presence of APBs, taking into account that APBs should be present between C nanodomains with different orientations. *x*(*M*2) ≠ 0 when the interatomic vectors *r* are mainly ‘intra-domain’ distances, while *x*(*M*2) averages to zero when *r* are mainly inter-domain distances. On increasing *x*_Gd_, the average dimension of the nanodomains increases and, at the same time, the APB concentration decreases. However, C nanodomains have been detected even for the *x*_Gd_ = 0.250 sample, whereas superstructure peaks are apparent only in the C* and C solid solutions.

The apparent inconsistency between the reciprocal- and real-space results can be understood as follows. Firstly, the effect of the Gd dopant in the CeO_2_ structure is modelled by defining *p* (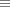 *x*_Gd_) as the site occupancy of Gd atoms in the cationic sites in Ce_1−*x*_Gd_*x*_O_2−*x*/2_ solid solutions. According to the percolation theory, the site percolation for a three-dimensional simple cubic lattice is *p*_C_ ≃ 0.311 (Martins & Plascak, 2003 ▸). This threshold is close to the *x*_Gd_ value at the F–C* boundary and ideally paves the way to include the percolation line of reasoning in the present investigation.

It should then be kept in mind that, by analysing the diffraction data in reciprocal space, each crystallite has to be considered as a whole. In fact, one single triplet of orthogonal axes is needed to describe its structure, and some simple rules relate the orientation of different C-type droplets/nano­domains to one another. In this sense, a crystallite that is compositionally inhomogeneous and exhibits a distribution of crystalline orientations on the nanometre scale differs from a mechanical mixture of fluorite and C-type nanopowders.

Since we are discussing the presence/absence of superstructure peaks as a function of the Gd concentration, in the following we will ignore the diffuse scattering contribution and focus only on the Bragg peaks. In a finite perfect crystal, the structure factor of a reflection **H** (where **H** is a point of the reciprocal lattice) can be written as 
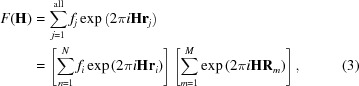
where the sum is intended to be over all the atoms in the sample. In equation (3), *f_j_* are the atomic scattering factors, *N* is the number of atoms/ions in the unit cell, *M* is the number of unit cells in the crystal and **R**_*m*_ is a point in the real-space lattice. *F*(**H**) depends only on the position of the *n* atoms within one unit cell.

In a defective real crystal, one can express the same structure factor as 
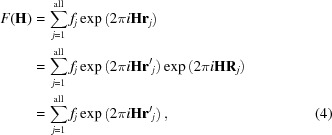
where **r**′_j_ = −**r**_*j*_ − **R**_*j*_ and **R**_*j*_ is the vector in real space needed to move atom *j* from its actual position in the crystal to the cell at the axes origin.

As stated above, the atomic positions in the C-type structure can be uniquely related to the fluorite atomic positions (see §3.1.1 and Table S1). As a consequence, **r**′_*j*_ can be rewritten as **r**′_*j*_ = **r** ^F^_*j*_ + δ**r**_*j*_, where **r** ^F^_*j*_ is the equilibrium position of ions in fluorite, while *δ* **r**_*j*_ is the displacement of the same site in the C-type arrangement. We can group together all ions which have the same **r** ^F^_*j*_ vector in the different *m* unit cells of the crystallite and rewrite the right-hand side of equation (4) as 
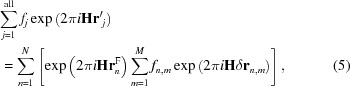
where *f*_*n*,*m*_ means that different atoms can occupy the same site in different cells. In equation (5), δ**r**_*n*,*m*_ can be interpreted as the resultant displacement of the *n*-th atom in the *m*-th cell and has the form 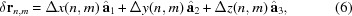
and 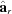 = **a**_*r*_/|**a**_*r*_| (*r* = 1, 2, 3), where **a**_*r*_ are the cell vectors.

Let us now work out the expected values 〈δ**r**_*n*_〉 of a set of δ**r**_*n*,*m*_ displacement vectors. The PDF analysis showed that the atomic positions within the droplets are quite close to their values in pure CeO_2_ and Gd_2_O_3_. We will therefore use the following approximation: the modulus of δ**r**_*n*,*m*_ is equated to the Gd_2_O_3_ value for Gd_2_O_3_-like droplets and set to zero for ions in CeO_2_-like droplets.

According to the previous assumptions, the displacement vectors pertinent to a given atomic position *n* may have six different directions obtained by either ‘locally’ permuting the crystallographic axes or inverting their orientations. In this way, six subsets of δ**r** ^*i*^_*n*,*m*_ values (*i* = 1…6) are naturally defined.

Starting from a given subset of displacement vectors δ**r** ^1^_*n*,*m*_ and applying the permutation operators **A**_1_, **A**_2_ and **A**_3_, the orientations of the remaining ones are obtained as follows 
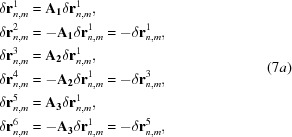
where 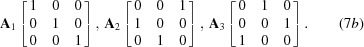
In other words, we are assuming a discrete probability distribution of displacement vectors to calculate the mean value 〈δ**r**_*n*_〉 over all possible δ**r**_*n*,*m*_ directions by multiplying each δ**r** ^*i*^_*n*,*m*_ by its probability *P*_*i*_. Summing up all these products, one obtains 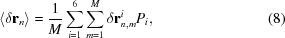
with 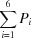 = *x*_Gd_. After suitably regrouping common terms, one can write equation (8) as 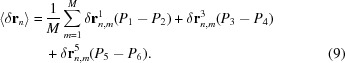
In this context we can distinguish between two main cases:

(i) If all six displacement directions are likely to appear in the same crystallite with the same probability, the mean displacement becomes 〈δ**r**_*n*_〉 = 0.

(ii) If *P*_1_ ≠ *P*_2_ and/or *P*_3_ ≠ *P*_4_ and/or *P*_5_ ≠ *P*_6_, then 〈δ**r**_*n*_〉 ≠ 0, and hence each sum 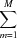 *f*_*n*,*m*_ exp(2π*i* **H**δ**r**_*n*,*m*_) ≠1 in equation (5). This accounts for the emergence of additional satellites in the powder diffraction pattern.

Using the above formalism it is possible to reconcile the reciprocal- and real-space findings throughout the whole compositional range of the solid solution. One starts by considering the fluorite solid solutions, *i.e. x*_Gd_ ≤ 0.25 (see Fig. 7 ▸ *c*). C-type nanodomains of various extents exist within each crystallite. They do not percolate and are embedded in the fluorite structure, leading to a progressive reduction and eventual vanishing of *x*(*M*2) while increasing *r* (see Fig. 5 ▸). Since the enthalpy of F–C surfaces should not depend on the orientation of the displacement vector, all six different distortion directions should occur in the same crystallite with the same probability and all the δ**r**_*n*,*m*_ vectors average to zero. For any *n*-th site, a distribution of atomic positions exists, the mean value of which is **r** ^F^_*n*_ (the same as for fluorite), and the extinction rules of the fluorite structure (*i.e.* of the 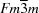 space group) apply. The *F*(**H**) values of the superstructure peaks average out to zero in the whole crystallite, as is experimentally evident for the *x*_Gd_ ≤ 0.250 samples. However, static disorder is given by a finite distribution of atomic equilibrium positions; this is expected to convolve with thermal vibrations, increasing the atomic mean-square parameters, in agreement with the huge value increase observed in the displacement parameters when *x*_Gd_ increases.

At *x*_Gd_ = 0.313, the percolation threshold is reached: a nanodomain with a given displacement vector orientation should percolate through the whole crystallite (see Fig. 7 ▸ *c*). This causes a symmetry break: the volumes of nanodomains with different orientations, averaged over the whole crystallite, no longer equate. The δ**r**_*n*,*m*_ values do not average to zero and superstructure peaks arise in the experimental patterns. As previously shown, superstructure peaks in the C* zone are broader than the structure peaks, and this seems to be consistent with both the presence of APBs and the finite volume-averaged dimension of the nanodomains.

Upon further increase in *x*_Gd_, the volume of percolating C-type domains increases and, for *x*_Gd_ ≥ 0.5, the APBs are negligible owing to the long-range correlation of the C-type phase: one of the six possible displacement directions *i* becomes predominant at the expense of the remaining ones.

In this case, focusing again on *x*(M2), equation (9) can be rewritten as: 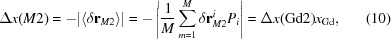
where Δ*x*(Gd2) = −0.0313 is the value of Δ*x*(*M*2) [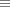 *x*(*M*2)] for pure Gd_2_O_3_. Equation (10) is displayed in Fig. 3 ▸(*a*) as a dashed blue line. It can be seen that in the ‘C’ zone this line is almost superimposed on the experimental data, underpinning the idea that one percolating C-type nanodomain permeates the whole crystallite with its coherence length.

The compositional evolution of ESR data can also be explained within the percolation framework. In the fluorite zone, the experimental curves seem to be the sum of different contributions: many C-type nanodomains of different dimensions give rise to distributions of Gd–Gd dipolar interactions coexisting in the same crystallite. With *increasing x*_Gd,_ Δ*H*_pp_ *increases* suddenly as a consequence of the increased number of Gd–Gd contacts on the long-range scale. At the percolation edge, at least one C-type nanodomain percolates along each crystallite and a Δ*H*_pp_ step of 800 Gauss wide is detected at the F–C* edge. When *x*_Gd_ increases further across the C*–C boundary, the C-type domains merge together to form one preponderant C-domain which starts to dominate the whole ESR signal.

## 5. Summary and conclusions   

We have presented a new bottom-up approach for investigating the structural disorder in solid solutions on different length scales, which can shed light on the relations between the short-range and the average structure of these materials through the analysis of disorder on the mesocopic scale.

This approach has been followed for the case of Ce_1−*x*_Gd_*x*_O_2−*x*/2_ solid solutions by means of real-space (PDF) and reciprocal-space (Rietveld refinement, WPPM and Williamson–Hall) analysis of XRPD data and ESR data processing. PDF analysis on a length scale of some tens of nanometres has been possible thanks to the outstanding *Q* resolution of the experimental setup of the ID31 beamline at the ESRF (now ID22).

The results obtained so far can be summarized as follows:

(i) In the shortest *r* range (less than 1 nm), all the Ce_1−*x*_Gd_*x*_O_2−*x*/2_ solid solutions can be suitably described using a biphasic model where both CeO_2_ and Gd_2_O_3_ ‘droplets’ coexist. Both |*x*(*M*2)| and Δ*d*(*MM*) values, which are fingerprints of ordering within the C-type phase, increase as a function of *x*_Gd_ up to *x*_Gd_ = 0.500, until they attain a saturation limit. When the biphasic model is applied to wider *r* ranges (up to ∼2 nm), *x*(*M*2) increases rapidly *versus r*, suggesting that the correlation length of pure Gd_2_O_3_ droplets is very short.

(ii) PDF analysis has been extended up to 40 nm by fitting data *via* a C-structure based model. For samples in the 0.25 ≤ *x*_Gd_ ≤ 0.433 interval, the slopes of curves plotting *x*(*M*2) *versus* *r* are positive; for *x*_Gd_ ≥ 0.50 the analogous curves are flat.

(iii) For *x*_Gd_ ≤ 0.25, the average structure is that of fluorite. With increasing *x*_Gd_ the structure turns into C-type but, for 0.313 ≤ *x*_Gd_ ≤ 0.433 (the so-called C* zone), the FWHMs of the superstructure peaks are wider than those of the structure peaks. This behaviour was modelled by both introducing extended defects such as APBs and considering the finite correlation length of C-type nanodomains.

As to the ESR results, Δ*H*_pp_ rises rapidly in the fluorite zone. At about 800 Gauss it displays a step-like behaviour, corresponding to the F–C* boundary, and increases smoothly for larger *x*_Gd_ values.

All the above results can be rationalized in the framework of a percolation-driven phase transition, since the site percolation threshold for a cubic lattice (*p*_C_ ≃ 0.311) is close to the *x*_Gd_ value at the F–C* boundary.

In the whole compositional range, the point defects, *i.e.* Gd dopant ions and O vacancies, cluster together to form C and F droplets. In fluorite solid solutions, the droplets assemble to form, in turn, C-type nanodomains of various sizes. They do not percolate and are embedded in the fluorite structure. The probabilities *P*_*i*_ associated with displacement vectors with different orientations are all the same and, on average, the ions have the same positions as in fluorite (Δ**r**_*n*,*m*_ values equalize to zero). For this reason, the structure factors *F*(**H**) of the superstructure peaks average to zero and the mean structure appears to be fluorite.

The percolation edge is reached at the F–C* boundary; a nanodomain with a given displacement orientation should percolate through the whole crystallite. This causes a symmetry break: the probabilities *P*_*i*_ and the volumes of nanodomains with different orientations, averaged over the whole crystallite, are no longer equal and the Δ**r**_*n*,*m*_ values differ on average from zero. Superstructure peaks appear in the experimental patterns. In the C* structure, a non-negligible number of APBs are present due to the interfaces between C-type nanodomains with different orientations.

When *x*_Gd_ is further increased, the volume of the percolating C-type domains enlarges and, crossing the C*–C boundary, one *P*_*i*_ value prevails (*P*_*i*_ ≃ *x*_Gd_). Accordingly, for *x*_Gd_ ≥ 0.5, the APB concentration is negligible, the widths of the structure and superstructure peaks are equal and *x*(*M*2) = *x*(Gd2)*x*_Gd_ [see equation (10)].

In this context, point defects, droplets and nanodomains can be considered as successive hierarchical levels of engrossing defect structures which gradually self-assemble to build up the long-range structure of Ce_1−*x*_Gd_*x*_O_2−*x*/2_ solid solutions.

It is worth noting that cation mobility is known to be very low in fluorite-structured oxides. On the one hand, this ensures that the cation distribution detected at 90 K is the same as that under operating conditions (800–1000 K). On the other hand, the temperature at which the samples are synthesized should influence the Gd distribution. We are thus planning to extend this analysis to selected samples annealed at higher *T* values.

This work has shown that extending the PDF analysis to spatial regions of some tens of nanometres allows one to reconcile structural findings at different length scales in Ce_1−*x*_Gd_*x*_O_2−*x*/2_ solid solutions, and also to identify structural fingerprints of disorder in the mesoscopic range, such as the compositional evolution of the *x*(*M*2) positional degree of freedom and the broadening of superstructure peaks.

We believe that the approach reported here may be suitably employed for the analysis of disorder in a broader class of highly doped materials.

## Supplementary Material

Figures and Tables not reported in the text. DOI: 10.1107/S2052252515011641/yu5007sup1.pdf
