# Supplementary material for: Percolating hierarchical defect structures drive phase transformation in Ce1−xGdxO2−x/2: a total scattering study
Source: IUCrJ. 2015 Jul 30;2(Pt 5):511–22. doi: 10.1107/S2052252515011641 (PMC4547819; doi:10.1107/S2052252515011641)
Supplement: Supplementary file 1 [file m-02-00511-sup1.pdf]

IUCrJ

Volume 2 (2015)

Supporting information for article:

Percolating Hierarchical Defect Structures drive Phase Transformation in Ce1-xGdxO2-x/2: a Total Scattering Study

Marco Scavini, Mauro Coduri, Mattia Allieta, Paolo Masala, Serena Cappelli, Cesare Oliva, Michela Brunelli, Francesco Orsini and Claudio Ferrero

Percolating hierarchical defect structures in $Ce_{1-x}Gd_xO_{2-x/2}$: a total scattering study

Marco Scavini[a,b], Mauro Coduri[a,c] , Mattia Allieta[a], Paolo Masala[a], Serena Cappelli[a], Cesare Oliva[a,b], Michela Brunelli[d], Francesco Orsini[e], Claudio Ferrero[f]

[a]Dipartimento di Chimica, Università di Milano, Via C. Golgi 19, I-20133 Milano, Italy

[b]CNR-ISTM, Ist. Sci. & Tecnol. Mol., I-20133 Milan, Italy

[c]Istituto per l'Energia e le Interfasi, CNR-IENI, C.so Promessi Sposi 29, 23900 Lecco, Italy

[d]S.N.B.L./E.S.R.F., 71, Avenue des Martyrs, CS 40220, 38043 Grenoble Cedex 9, France[e]Dipartimento di Fisica, Università di Milano, Via G. Celoria 19, I-20133 Milano, Italy

[f]E.S.R.F.-The European Synchrotron, 71, Avenue des Martyrs, CS 40220, 38043 Grenoble Cedex 9, France

Figure S1

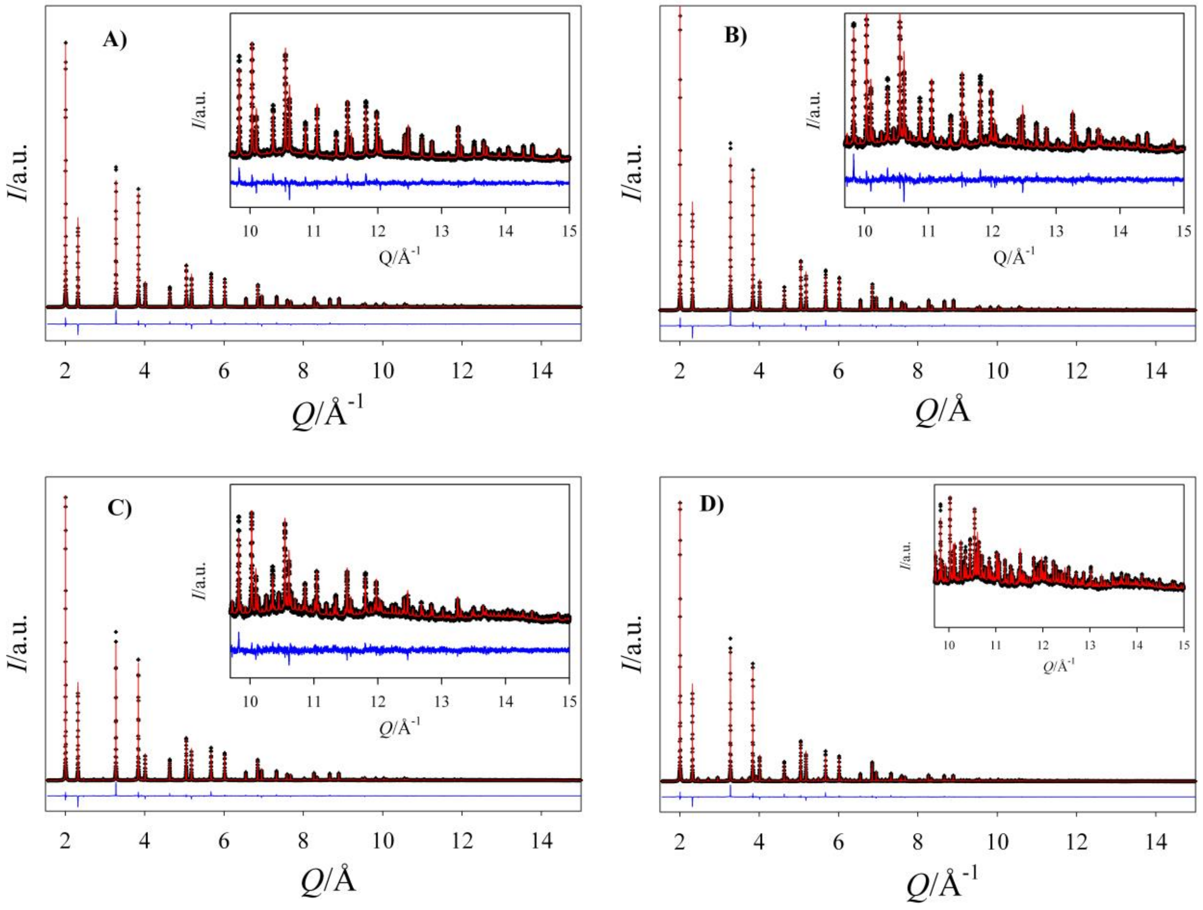

Figure S1 XRPD patterns of $Ce_{1-x}Gd_xO_{2-x/2}$ samples and Rietveld refinements obtained using the structural models described in Table S1. A) x_{Gd}=0.313; B) x_{Gd}=0.344; C) x_{Gd}=0.375; D) x_{Gd}=0.438. Measured (black crosses) and calculated (red lines) profiles are shown as well as fit residuals (blue lines). The insets highlight the diffraction profiles in the high Q regions

Figure S2

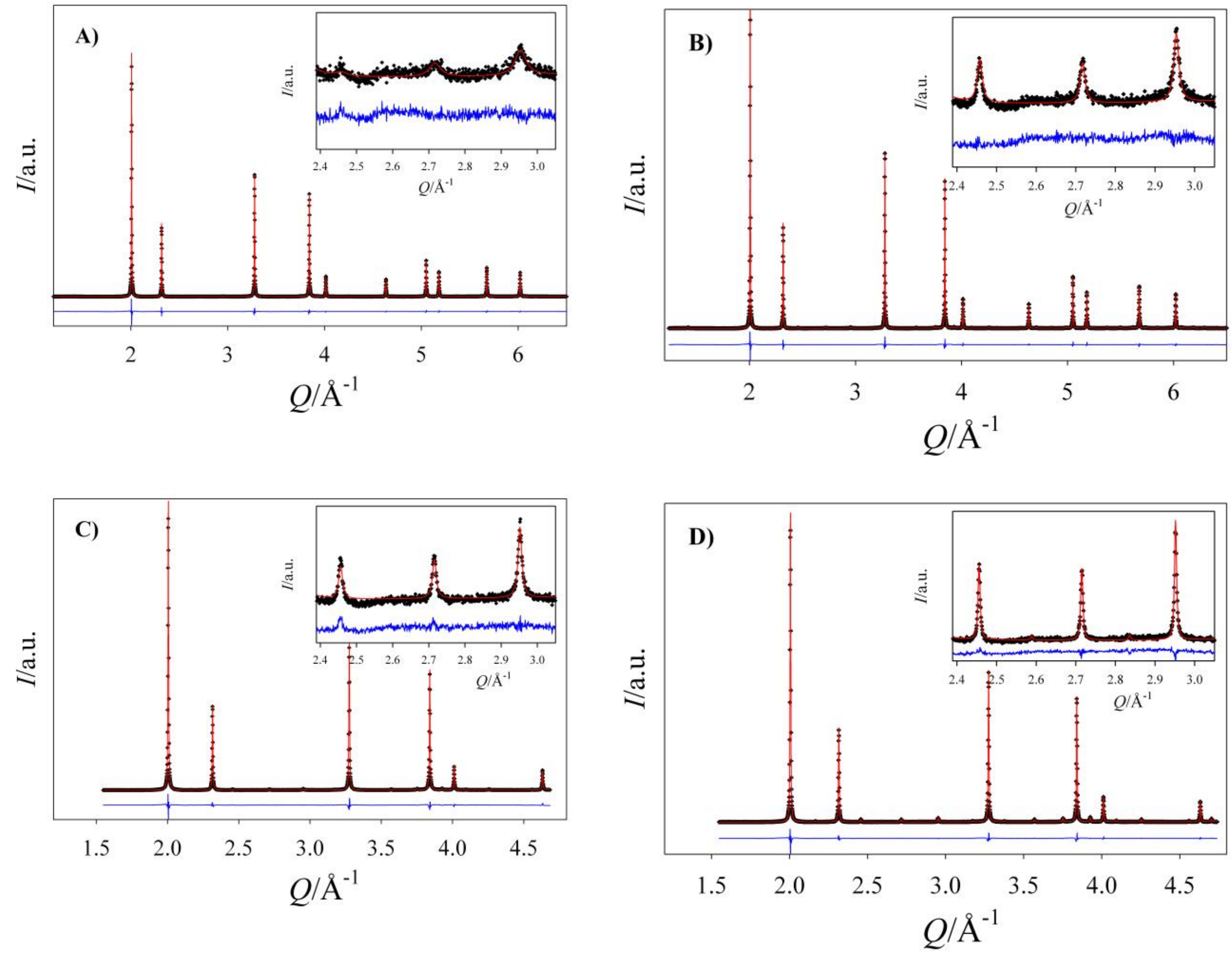

Figure S2 Same XRPD patterns as in Figure S1 using the WPPM approach. A) x_{Gd}=0.313; B) x_{Gd}=0.344; C) x_{Gd}=0.375; D) x_{Gd}=0.438. Measured (black crosses) and calculated (red lines) profiles are shown as well as fit residuals (blue lines). The insets highlight the diffraction profiles in the Q regions corresponding to the most intense superstructure peaks

Sites	C-type	Shifted C-type	C-type with shifted origin	2×2×2 Fluorite	1×1×1 Fluorite
**M1**	8b: ¼, ¼, ¼	¼, ¼, ¼	¼, ¼, 0	¼, ¼, 0	4a: ½, ½, 0
**M2**	24d *x*,0, ¼	Δ*x*(M2), 0, ¼	Δ*x*(M2), 0, 0	0, 0, 0	4a: 0, 0, 0
**O1**	48e *x*,*y*,*z*	3/8+Δ*x*(O1),1/8+Δ*y*(O1), 3/8+Δ*z*(O1)	3/8+Δ*x*(O1), 1/8+Δ*y*(O1), 1/8+Δ*z*(O1)	3/8, 1/8, 1/8	8c: ¾, ¼, ¼
**O2**	16c *x*,*x*,*x*	3/8+Δ*x*(O2), 3/8+Δ*x*(O2), 3/8+Δ*x*(O2).	3/8+Δ*x*(O2), 3/8+Δ*x*(O2), 1/8+Δ*x*(O2).	3/8, 3/8, 1/8	8c: ¾, ¾, ¼

Table S1. **Scheme of the relationship between fluorite and C-type phases**.

Column-wise legend: **C-type**: atom positions and corresponding Wyckoff symbols for the sites in the C-type structure; **Shifted C-type:** same setting as the previous column, with addition of the Δ values with respect to special positions; **C-type with shifted origin:**: the cell origin is shifted with respect to the previous column by <0, 0, ¼>; **2×2×2 Fluorite:** same as the previous column with all Δ values set to zero to recover the fluorite structure; **1×1×1 Fluorite:** atom positions and corresponding Wyckoff symbols for the sites in the fluorite structure.

Sample	x_{Gd}=0.313	x_{Gd}=0.344	x_{Gd}=0.375	x_{Gd}=0.438
Space group	*Ia*-3	*Ia*-3	*Ia*-3	*Ia*-3
a/Å	10.84730(2)	10.84760(5)	10.85483(6)	10.85488(5)
x_{M2}	-0.00373(5)	-0.00548(5)	-0.00820(5)	-0.01309(3)
x_{O1}	3/8	3/8	0.376(1)	0.3778(5)
y_{O1}	1/8	1/8	0.135(1)	0.1412(5)
z_{O1}	3/8	3/8	0.378(2)	0.3774(8)
x,y,z_{O2}	3/8	3/8	0.377(4)	0.3775(2)
U_{11}/Uiso(M1)	0.012(1)	0.016(1)	0.0178(9)	0.02341(4)
U_{12}(M1)	------	0.0105(8)	0.0058(9)	0.0122(4)
U_{11}/Uiso(M2)	0.0114(3)	0.0082(6)	0.0094(4)	0.0077(1)
U_{22}(M2)		0.016(1)	0.017(1)	0.0137(4)
U_{33}(M2)	------	0.015(2)	0.016(1)	0.0150(5)
U_{23}(M2)	------	-0.0069(6)	-0.0103(7)	-0.0116(2)
U_{iso}(O1/O2)	0.0191(3)	0.0215(4)	0.0209(6)	0.0184(6)
R(F^2)	0.0356	0.0485	0.0453	0.0609
R_P	0.0452	0.0479	0.0466	0.0418

Table S2. Rietveld refinement results referring to C-type $Ce_{1-x}Gd_xO_{2-x/2}$ samples with x_{Gd}=0.313, 0.344, 0.375 and 0.438. Fractional atomic coordinates are dimensionless, while the mean square displacements U_{iso} and U_{ij} are expressed in Å^2. The estimated standard deviations are in brackets.

Best fit of PDF data at short *r* range using the monophasic and the biphasic models

The PDF experimental profiles were best fitted using either the monophasic or the biphasic model (see Figure 4 in the text).

As to the *monophasic model*, the C-type structure was assumed (space group *Ia*-3) for all the samples in the C* zone, varying the cell constant, the *x*(M2) coordinate and three different isotropic *msd* parameters for M1, M2 and O1/O2 positions. The refined parameters are reported in Table S2a.

As to the *biphasic model*, coexisting CeO_2 (*Fm*-3*m*) and Gd_2O_3 (*Ia*-3) were allowed for, keeping site occupations and O coordinates fixed to pure ceria and gadolinia values. In order to avoid over-parameterization and consequent parameter correlations, in the *biphasic model* only two cell parameters, the phase fractions, the *x*(M2) coordinates, one *msd* parameter for all cationic sites and another *msd* parameter for all the anionic sites in the two phases were allowed to vary. The optimized parameters are reported in Table S2b.

Sample	x_{Gd}=0.313	x_{Gd}=0.344	x_{Gd}=0.375	x_{Gd}=0.438
Space group	*Ia-3*	*Ia-3*	*Ia-3*	*Ia-3*
a/Å	10.7248(8)	10.7064(7)	10.7002(8)	10.6492(5)
x_{M2}	-0.0003(2)	-0.0005(2)	-0.0063(2)	-0.0042(1)
U_{iso}(M1)	0.0255(6)	0.02916(6)	0.0190(8)	0.0060(1)
U_{iso}(M2)	0.00418(6)	0.00590(6)	0.0058(1)	0.00898(7)
U_{iso}(O)	0.0558(8)	0.0578(6)	0.067(1)	0.022(1)
Rw	0.218	0.233	0.268	0.303

Table S2a. Real space Rietveld refinement (based on the monophasic model) of the calculated G(*r*) curves in the $1.5 \leq r \leq 6.1$ Å range. The fractional atomic coordinates are dimensionless, while the *msd* U_{iso} are expressed in Å^2. The estimated standard deviations are in brackets.

Sample	x_{Gd}=0.313	x_{Gd}=0.344	x_{Gd}=0.375	x_{Gd}=0.438
Fluorite phase: space group *Fm-3m*				
a/Å	5.3729(6)	5.3638(4)	5.3593(5)	5.3450(3)
% .fluorite phase	68.2(3)	63.9(2)	61.9(3)	57.0(1)
C-type phase: space group *Ia-3*				
a/Å	11.060(3)	11.039(2)	11.047(3)	11.040 (1)
x_{M2}	-0.0275(2)	-0.0284(1)	-0.0293(2)	-0.03136(6)
%.C-type phase	31.8(3)	36.1(2)	38.1(3)	43.0(1)
Common parameters				
U_{iso}(M)	0.00566(5)	0.00575(5)	0.00590(6)	0.00607(3)
U_{iso}(O)	0.0534(8)	0.0487(8)	0.051(1)	0.0578(6)
Rw	0.096	0.091	0.097	0.118

Table S2b. Real space Rietveld refinement (based on the biphasic model) of the calculated G(*r*) curves in the $1.5 \leq r \leq 6.1$ Å range. The fractional atomic coordinates are dimensionless, while the *msd* U_{iso} are expressed in Å^2. The estimated standard deviations are in brackets
